# Clinical Risk Factors of Licorice-Induced Pseudoaldosteronism Based on Glycyrrhizin-Metabolite Concentrations: A Narrative Review

**DOI:** 10.3389/fnut.2021.719197

**Published:** 2021-09-17

**Authors:** Tetsuhiro Yoshino, Saori Shimada, Masato Homma, Toshiaki Makino, Masaru Mimura, Kenji Watanabe

**Affiliations:** ^1^Center for Kampo Medicine, Keio University School of Medicine, Tokyo, Japan; ^2^Department of Pharmaceutical Sciences, Division of Clinical Medicine, Faculty of Medicine, University of Tsukuba, Tsukuba, Japan; ^3^Department of Pharmacognosy, Graduate School of Pharmaceutical Sciences, Nagoya City University, Nagoya, Japan; ^4^Department of Psychiatry and Neurology, Keio University School of Medicine, Tokyo, Japan

**Keywords:** Japanese Kampo medicine, licorice, pseudoaldosteronism, hypokalemia, pharmacokinetics

## Abstract

Licorice, the dried root or stolon of *Glycyrrhiza glabra* or *G. ularensis*, is commonly used worldwide as a food sweetener or crude drug. Its major ingredient is glycyrrhizin. Hypokalemia or pseudoaldosteronism (PsA) is one of the most frequent side effects of licorice intake. Glycyrrhizin metabolites inhibit type 2 11β-hydroxysteroid dehydrogenase (11βHSD2), which decomposes cortisol into inactive cortisone in the distal nephron, thereby inducing mineralocorticoid receptor activity. Among the several reported glycyrrhizin-metabolites, 18β-glycyrrhetyl-3-*O*-sulfate is the major compound found in humans after licorice consumption, followed by glycyrrhetinic acid. These metabolites are highly bound to albumin in blood circulation and are predominantly excreted into bile via multidrug resistance-associated protein 2 (Mrp2). High dosage and long-term use of licorice are constitutional risk factors for PsA. Orally administered glycyrrhizin is effectively hydrolyzed to glycyrrhetinic acid by the intestinal bacteria in constipated patients, which enhances the bioavailability of glycyrrhizin metabolites. Under hypoalbuminemic conditions, the unbound metabolite fractions can reach 11βHSD2 at the distal nephron. Hyper direct-bilirubin could be a surrogate marker of Mrp2 dysfunction, which results in metabolite accumulation. Older age is associated with reduced 11βHSD2 function, and several concomitant medications, such as diuretics, have been reported to affect the phenotype. This review summarizes several factors related to licorice-induced PsA, including daily dosage, long-term use, constipation, hypoalbuminemia, hyper direct-bilirubin, older age, and concomitant medications.

## Introduction

**Figure 1:**
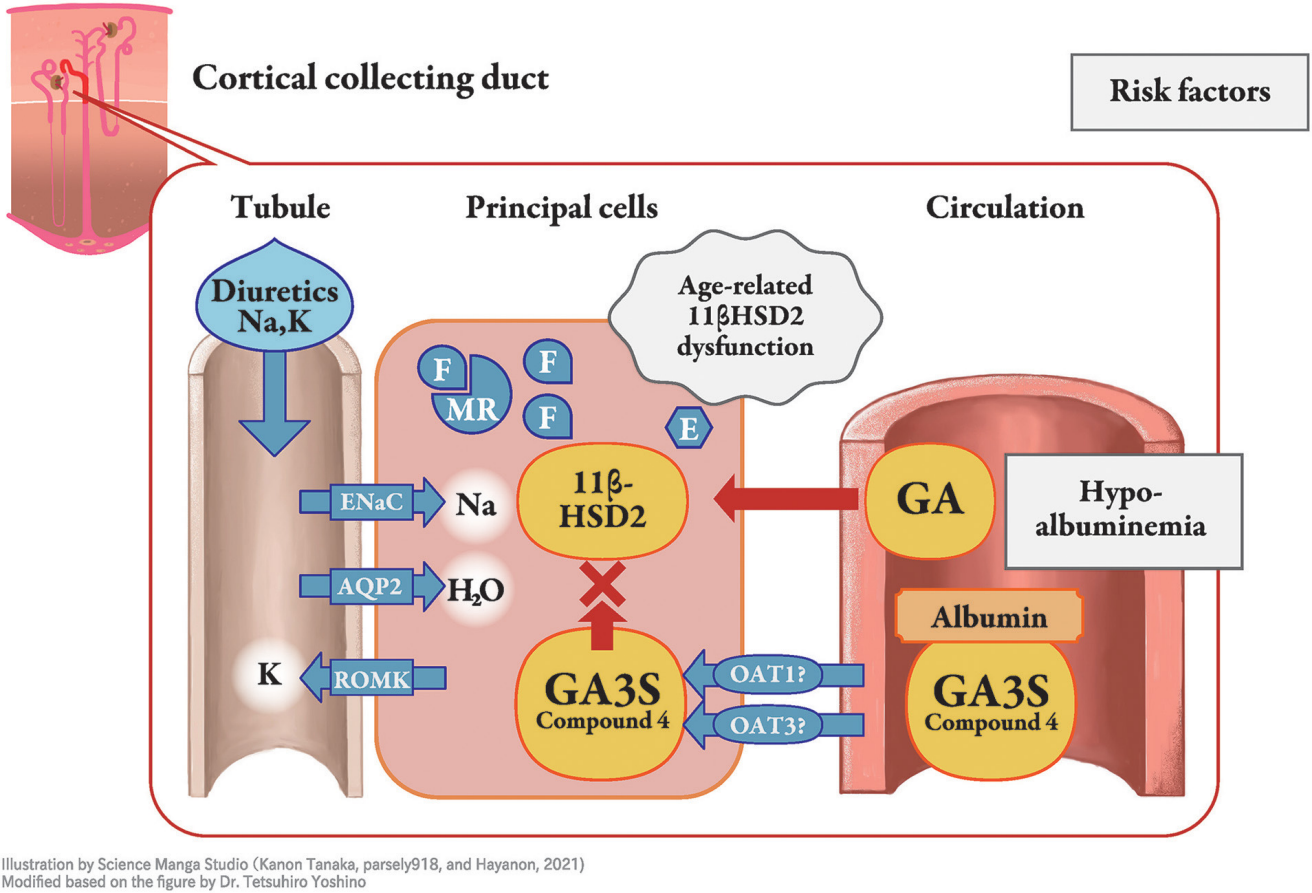
Molecular mechanism of licorice-induced pseudoaldosteronism. At the distal nephron, especially in the principal cells of the cortical collecting duct, type 2 11β-hydroxysteroid dehydrogenase (11β-HSD2) is inhibited by glycyrrhetinic acid-3-*O*-sulphate (GA3S) and possibly other metabolites, resulting in increased levels of cortisol (F), with similar affinity to the mineralocorticoid receptor as that of for aldosterone. Mineralocorticoid receptors (MR) induce stabilization of epithelial sodium channels (ENaC) on the apical side, which increases sodium reabsorption. This results in peripheral edema, hypertension, and lower plasma renin activity, whereas potassium is excreted as a cationic ion via the renal outer medullary potassium channel. The unbound fraction of metabolites under hypoalbuminemia enables them to efficiently reach 11β-HSD2. Older age is related to lower function of 11β-HSD2, and diuretics affect their phenotype. E, cortisone; AQP, aquaporin; ROMK, renal outer medullary potassium channel; GA, glycyrrhetinic acid; OAT, organic anion transporter.

Licorice, the dried root or stolon of *Glycyrrhiza glabra* or *G. ularensis*, is commonly used as a food sweetener or crude drug worldwide. Wide-range pharmaceutical effectiveness of licorice includes anti-ulcer, anti-spasmodic, anti-inflammatory, anti-oxidative, anti-virus, anti-microbial, anti-carcinogenic, and anti-androgenic properties (1). Pseudoaldosteronism (PsA) is, however, one of the most frequent side effects of licorice intake and has been well described by Conn et al. (2). The clinical presentation of PsA is similar to that of primary aldosteronism and is characterized by peripheral edema, hypertension, laboratory hypokalemia, and lower plasma renin activity, due to the excessive action of mineralocorticoid receptors. The mineralocorticoid receptor stabilizes epithelial sodium channels on the apical side of the cortical collecting duct principal cell, which increases sodium reabsorption, corresponding to peripheral edema, hypertension, and lower plasma renin activity (Figure 1), whereas potassium is excreted as a cationic ion via the renal outer medullary potassium channel, which results in hypokalemia and, in severe cases, myopathy or arrhythmia (1). Most cases of licorice-induced PsA are self-limiting and are resolved once licorice intake ceases, without any specific treatment. However, some cases can progress to severe hypokalemia and life-threatening arrhythmia (3).

## Pathophysiology of Licorice-Induced PsA

Licorice contains glycyrrhizin (GL), a glycoside of glycyrrhetinic acid (GA) containing two molecules of glucuronic acid. Licorice has long been known to exert corticosteroid-like action, and GL and GA are considered to cause PsA by binding to mineralocorticoid receptors (2). However, their affinity for the receptor is significantly lower than that of the original substrate, aldosterone (4, 5), and the hypothesis that it acts by directly binding to the receptor at the actual blood concentrations of GL and GA is unrealistic (1). Furthermore, previous studies found that licorice-induced PsA did not occur in patients and animals with adrenal deficiency who had lower blood cortisol levels (6–10).

Cortisol, an adrenocortical hormone, has the same affinity for mineralocorticoid receptors as aldosterone; however, it occurs at a higher concentration in the blood. Cortisol is then decomposed by type 2 11β-hydroxysteroid dehydrogenase (11βHSD2) in renal tubule cell cytoplasm into cortisone, which has a lower affinity for the receptor (11), especially at the distal nephron (12, 13) in the normal state. Inhibition of 11βHSD2 by GL metabolites, rather than direct binding of GL or its metabolites to mineralocorticoid receptors was thus considered as the mechanism of licorice-induced PsA (14–16). With GL metabolites in the 11βHSD2-expressing cells, inhibition of 11βHSD2 results in a higher concentration of cortisol that binds to and stimulates the mineralocorticoid receptor (Figure 1). Therefore, the difference between primary aldosteronism and PsA is the plasma aldosterone concentration, which is lower in PsA and higher in primary aldosteronism. The aldosterone concentration is suppressed by negative feedback in licorice-induced PsA, even though aldosterone metabolism in the liver could be suppressed by GL or GA (17).

There are several other causes of PsA, including enzymatic defects in adrenal steroidogenesis (deficiency of 17α-hydroxylase and 11β-hydroxylase), gain-of-function mutations in the mineralocorticoid receptor (18), saturation of mineralocorticoid receptor binding by cortisol (Cushing syndrome), alterations in 11βHSD2 (syndrome of apparent mineralocorticoid excess), and genetic alterations in sodium channel expression (Liddle syndrome) or the sodium-chloride co-transporter (Gordon syndrome) (19).

## GL-Metabolites That Truly Reach and Inhibit Type 2 11β-Hydroxysteroid Dehydrogenase

When GL is administered orally, it cannot be absorbed in its original form owing to its molecular structure, which contains both hydrophobic and hydrophilic parts. Orally ingested GL is hydrolyzed to GA by the intestinal bacteria (20–24), and GA appears as the main metabolite in blood circulation (20). Both GA and GL inhibit 11βHSD2 *in vitro*, but the inhibitory activity of GA is approximately 200 times higher than that of GL (15). Further, as GL does not appear in blood after licorice ingestion, GA has been considered as the causative agent for the onset of licorice-induced PsA that inhibits 11βHSD2 in humans. However, the plasma concentration of 3-monoglucuronyl glycyrrhetinic acid (3MGA) was reported to be significantly higher in patients with hypokalemia than in those with normokalemia and chronic hepatitis who had been orally treated with GL for more than four weeks, even though the plasma concentration of GA did not differ between the two groups (25). When GL is injected intravenously into rats, it is partially metabolized to 3MGA in the liver by lysosomal β-D-glucuronidase, after which GL and 3MGA are excreted with bile (26). Although 3MGA did not appear in the blood circulation and urine of normal Sprague-Dawley rats that were orally treated with GA, it was found in the blood and urine of multidrug resistance-associated protein (Mrp) 2-deficient Eisai hyperbilirubinuric rats (EHBR) (27). Both GA and 3MGA have high affinity for albumin (28–31); however, only 3MGA is the substrate of organic anion transporter (OAT) 1 and OAT3, which are expressed at the basolateral membrane of renal tubular epithelial cells (31). As a substrate of basolateral transporters, 3MGA is compatible with the intracellular space where 11βHSD2 is located, and is considered a causative agent of PsA. However, 3MGA has not been detected by mass spectrometry in humans after licorice intake (32–34). Instead, we isolated and identified 22α-hydroxy-18β-glycyrrhetyl-3-*O*-sulfate-30-glucuronide (compound 1), 22α-hydroxy-18β-glycyrrhetyl-3-*O*-sulfate (compound 2), 18β-glycyrrhetyl-3-*O*-sulfate (compound 3, GA3S), and 18β-glycyrrhetyl-3-*O*-sulfate-30-glucuronide (compound 4) as other GL metabolites in the urine of EHBR orally treated with GA (34–36). We also found that the blood and urinary concentrations of these new metabolites were much higher than those of 3MGA in EHBR orally treated with GA, and that their pharmacokinetic behavior was similar to that of 3MGA (34, 35). In humans, GA3S was detected at the highest concentration in the blood of patients with PsA who developed rhabdomyolysis due to licorice (34). Further, there were no cases where compound 1 was detected, while compound 2, GL, and 3MGA were rarely detected. The concentration of GA3S was still the highest, followed by GA and compound 4 in the serum of patients in a multicenter retrospective case series of milder PsA (33, 36). Therefore, we considered GA3S to be the most likely causative agent of PsA.

## Diagnosis of Licorice-Induced PsA

There is no concrete diagnostic criterion for licorice-induced PsA, and diagnosis is purely based on the clinical presentation of patients during licorice intake rather than by measuring the GL metabolite accumulation in blood. Therefore, diagnosing PsA is challenging, especially in mild cases. The serum concentrations of GA3S, GA, and compound 4 were found to be negatively correlated with serum potassium concentration, plasma renin activity, and aldosterone concentration (33, 36). From these results, it is suggested that such GL-metabolites could be used as an objective laboratory marker of “licorice-induced” PsA, and could be applied to the early detection and prevention of licorice-induced PsA especially in high risk patients.

As it is difficult to diagnose PsA based only on physical examination, including peripheral edema and hypertension, laboratory testing of parameters such as potassium and aldosterone levels is needed. The grade or occurrence of peripheral edema and hypertension in patients who consumed licorice did not correlate well with laboratory abnormalities, such as hypokalemia, low renin activity, aldosterone concentration, and GL metabolites (33, 36). Several studies have shown the expression of mineralocorticoid receptor and 11βHSD1/2 in the heart and arteries (37–39). Inhibition of 11βHSD2 resulted in an increased contractile response to phenylephrine (40) and altered endothelium-dependent relaxation of arteries due to decreased endothelial nitric oxide and increased endothelin-1 (41). These observations suggest that renal sodium reabsorption cannot fully explain hypertension in licorice-induced PsA.

## Clinical Risk Factors for Licorice-Induced PsA

### Pharmacokinetics

#### Daily Dosage

The daily dosage of licorice is a reasonable risk factor for licorice-induced PsA (42, 43). Of the 147 oral Kampo medicinal products for ethical use in Japan, 109 (74%) contain extracts with 1.0–8.0 g licorice for daily dose. Their package inserts describe hypertension, hypokalemia, arrhythmia, and rhabdomyolysis as adverse events of licorice-induced PsA. Kampo medicinal products containing more than or equal to 2.5 g licorice daily are contraindicated in patients with aldosteronism, myopathy, or hypokalemia, and those containing <2.5 g licorice daily still indicate these PsA-related conditions as potential side effects. As 74% of Kampo medicinal products contain GL, the use of multiple Kampo medicinal products can result in excessive GL ingestion, especially if the medicines are prescribed in multiple hospitals and clinics simultaneously.

#### Long-Term Use

Long-term use (>30 days) is also an important factor for developing licorice-induced PsA and related symptoms. In a retrospective cohort study, more than 80% of patients developed licorice-induced PsA and related symptoms, including hypokalemia in elderly patients (>60 years old) who received shakuyakukanzoto, which contains 6.0 g licorice, for longer than 30 days (44). This observation in the study is reasonable as the inhibition of 11βHSD plateaued after 2 weeks of GL ingestion (45).

#### Absorption

##### Constipation

**Figure 2:**
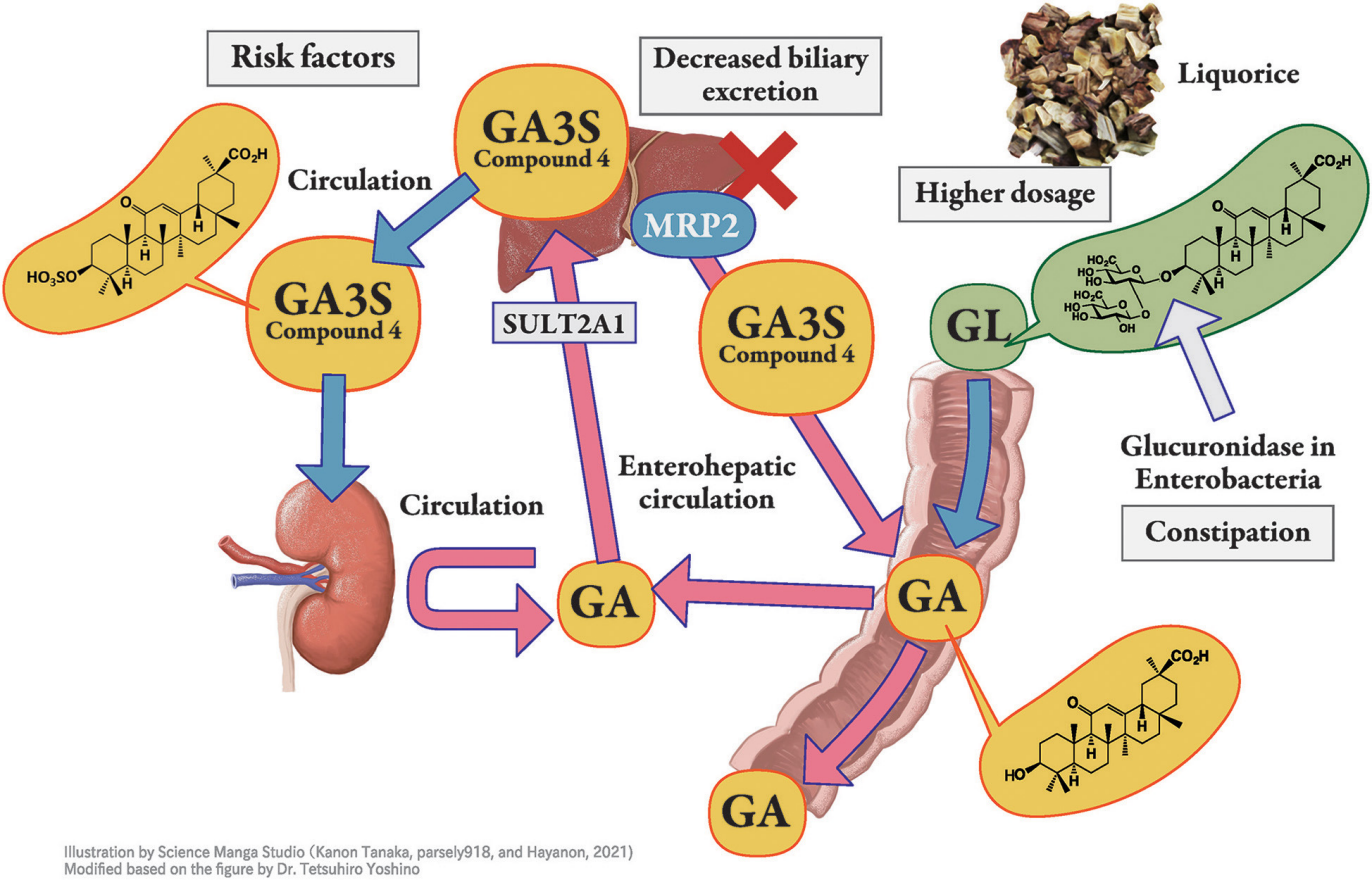
Pharmacokinetics of glycyrrhizin-metabolites. Licorice, the root or stolon of Glycyrrhiza glabra or G. ularensis, contains glycyrrhizin (GL), a glycoside of glycyrrhetinic acid (GA) with two molecules of glucuronic acid. When GL is administered orally, it is hydrolyzed to GA by the intestinal bacteria and then absorbed into blood circulation. GA in blood circulation is not excreted into the urine but is transferred into the liver, sulfated to GA-3-*O*-sulphate (GA3S) by sulfotransferase (SULT) 2A1, and excreted into bile via multidrug resistance-associated protein (MRP) 2. In humans, GA3S is detected at a higher concentration than GA. In the intestine, GA3S is hydrolyzed to GA by the intestinal bacteria and is partially absorbed from the intestine into blood circulation to exhibits enterohepatic circulation, whereas the unabsorbed portion of GA3S is excreted in the feces. Orally taken GL is effectively hydrolyzed to GA by intestinal bacteria in constipated patients, which enhances its bioavailability. Hyper direct-bilirubin could thus be a surrogate marker of multidrug resistance-associated protein 2 dysfunction.

When GL is administered orally, it is hydrolyzed to GA by the intestinal bacteria (20) and then absorbed into blood (Figure 2). GA3S, a sulfate conjugate of GA which is excreted via bile, is also hydrolyzed to GA by the intestinal bacteria and is then partially absorbed from the intestine into the blood circulation to exhibit enterohepatic circulation. The unabsorbed portions of GA and GA3S are then excreted in the feces. Thus, large individual variations in the blood concentration of GA were found even at the same dosage of GL or licorice. This variation may be due to the difference in the activity of hydrolyzed GL in the intestinal bacterial flora among the subjects. The hydrolyzation ratio also depends on the residence time of GL in the intestinal tract. The longer GL stays in the intestinal tract, the more it is hydrolyzed by bacterial β-glucuronidase, resulting in a higher serum concentration of GA (46).

#### Distribution

As GL metabolites are highly bound to serum albumin (>99.9%) in blood circulation (47, 48), they are not excreted in urine through glomerular filtration. However, they can be transported from blood circulation into tubular cells via OAT1 and OAT3, or other transporters and can participate in the inhibition of intracellular 11βHSD2 as well as their unbound forms (33–35). Hypoalbuminemia increases the unbound fraction of metabolites in blood circulation (30), resulting in enhanced distribution into principal cells where 11β-HSD2 is located (Figure 1). Therefore, hypoalbuminemia may be an important risk factor for PsA. Indeed, hypoalbuminemia has been identified as a risk factor in patients receiving yokukansan, which contains 1.5 g of licorice daily (49), as well as in other case series (50, 51).

#### Metabolism

To the best of our knowledge, there are no reports describing an individual variety of primary or secondary metabolism of GA in humans. We have previously reported that GA is conjugated by human liver sulfotransferase 2A1 (dehydroepiandrosterone sulfotransferase) into GA3S at C-3 (Figure 2). As serum concentrations of GA and GA3S correlated well, regardless of sex, sulfate conjugation is suggested to have a limited impact on inter-individual variation in GA pharmacokinetics (33).

#### Excretion

When bile excretion of GA3S is suppressed due to Mrp2 dysfunction (Figure 2), GA3S is transferred into the blood circulation. We have previously highlighted the involvement of Mrp2 in the biliary excretion of GL metabolites in rats (27, 34, 35) and possibly in humans (50, 51). GL metabolites significantly accumulate in the EHBR (27, 34, 35). While this phenomenon is still controversial in humans, we reported the co-occurrence of hypokalemia and hyperbilirubinemia, which could be a surrogate marker for MRP2 dysfunction in several cases; further, hypokalemia was found to be more common in patients with hyperbilirubinemia (50, 51). We consider hyper direct-bilirubin as a rare risk factor for licorice-induced PsA when compared with hypoalbuminemia, which is more common in outpatient settings (51).

### Other Factors

#### Age

Older age may affect several processes of GL metabolism, and licorice-induced PsA is common in elderly patients (43, 52). As age-dependent decrease in 11 βHSD2 activity in hypertensive patients has been reported (53), alteration in enzyme activity in aging is another risk factor for elderly subjects (Figure 1). In addition, elevation of serum cortisol concentrations occur in elderly which may be associated with decreased negative feedback at the hippocampus related to decreased glucocorticoid receptor concentration (54). This phenomenon could result in the inhibition of 11βHSD2 and also explains why licorice-induced PsA is common among elderly patients. To the best of our knowledge, there is no case report of licorice-induced PsA in pediatric patients meaning that licorice and GL is safe to use in children.

#### Female Sex

Several other factors have been proposed as risk factors for licorice-induced PsA, including female sex (55, 56), lower body weight (43), and reduced body surface area (57), which have not been well explained and might be confounding factors (52). Theoretically, constipation is common in female patients, and the higher dosage administered despite lower body weight and surface area compared with that in male patients even with the same daily dosage of licorice.

#### Concomitant Use of Medications

Concomitant use of medications, including antihypertensives, especially thiazide, loop diuretics, aldosterone blockers, angiotensin-converting enzyme inhibitors, and angiotensin receptor blockers, affects the phenotype of PsA. For example, concomitant use of potassium-losing diuretics increases the risk of hypokalemia in patients treated with yokukansan, an extract prepared with 1.5 g licorice (43, 49). Loop diuretics block Na-K-2Cl cotransporters, and thiazides block Na-Cl transporters in the distal nephron. Although these diuretics can prevent peripheral edema and hypertension, they increase intraductal flow into the collecting duct and stimulate sodium reabsorption (58), which increases potassium excretion and induces hypokalemia in patients taking licorice-containing products. Systemic glucocorticoid use can also aggravate the inhibition of 11βHSD2 that is similar to Cushing syndrome.

Conversely, potassium-sparing medications such as aldosterone blockers, angiotensin-converting enzyme inhibitors, and angiotensin receptor blockers prevent licorice-induced hypokalemia. Concomitant use of these medications thus hinders the early detection of licorice-induced PsA.

#### Dementia

Dementia also makes early detection of PsA difficult (43). Therefore, medical specialists, including pharmacists and nurses, should be aware of hypertension or peripheral edema in the patient, even though these physical signs are not very sensitive or specific for licorice-induced PsA.

## Conclusions

We summarized the current understanding regarding the pathophysiology of licorice-induced PsA and listed several factors that affect the pharmacokinetics of GL metabolites, including the daily dosage, dosing period, constipation, hypoalbuminemia, and hyperdirect-bilirubin. Further, older age and several concomitant medications are risk factors for licorice-induced pseudoaldosteronism. Importantly, a deep understanding of the crude drugs in Kampo preparations and the pathophysiology of licorice-induced PsA is necessary for the prevention and early diagnosis of PsA. Clinicians should be aware of PsA and balance the merit and demerit of using licorice or GL for therapy.

## Author Contributions

TY, SS, MH, and TM wrote the draft of the manuscript. MM and KW designed the study and revised the manuscript. All authors read and approved the final manuscript.

## Funding

This work was supported by a Grant-in-Aid from the Research Project for Improving Quality in Healthcare and Collecting Scientific Evidence on Integrative Medicine from AMED under grant numbers JP17lk0310036h0001, JP18lk0310049h0001, and JP19lk0310064h0001. The authors declare that this study received funding from Tsumura Co. In addition, TM received research grant supports from Tsumura & Co., Kracie Pharmaceuticals, JPS Pharmaceuticals, and Taisho Pharmaceutical Holdings. MM received research grant supports from Tsumura & Co. and Kracie Pharmaceuticals. The funders were not involved in the study design, collection, analysis, interpretation of data, the writing of article, or the decision to submit it for publication. An article processing charge, English editing, and figure drawing fee of this article were payed by the joint research program fund of Keio University and Tsumura Co.

## Conflict of Interest

TY is employed for the joint research program with Tsumura & Co. KW and MH received lecture fee from Tsumura & Co. The authors declare that the research was conducted in the absence of any commercial or financial relationships that could be construed as a potential conflict of interest. The handling editor declared a past collaboration with several of the authors TY, TM, and KW at the time of review.

## Publisher's Note

All claims expressed in this article are solely those of the authors and do not necessarily represent those of their affiliated organizations, or those of the publisher, the editors and the reviewers. Any product that may be evaluated in this article, or claim that may be made by its manufacturer, is not guaranteed or endorsed by the publisher.
